# Isolation of High-Purity Extracellular Vesicles by Extracting Proteins Using Aqueous Two-Phase System

**DOI:** 10.1371/journal.pone.0129760

**Published:** 2015-06-19

**Authors:** Jongmin Kim, Hyunwoo Shin, Jiyoon Kim, Junho Kim, Jaesung Park

**Affiliations:** 1 Department of Mechanical Engineering, Pohang University of Science and Technology, Pohang, Gyeong-buk, Republic of Korea; 2 School of Interdisciplinary Bioscience and Bioengineering, Pohang University of Science and Technology, Pohang, Gyeong-buk, Republic of Korea; University of South Florida College of Medicine, UNITED STATES

## Abstract

We present a simple and rapid method to isolate extracellular vesicles (EVs) by using a polyethylene glycol/dextran aqueous two-phase system (ATPS). This system isolated more than ~75% of melanoma-derived EVs from a mixture of EVs and serum proteins. To increase the purity of EVs, a batch procedure was combined as additional steps to remove protein contaminants, and removed more than ~95% of the protein contaminants. We also performed RT-PCR and western blotting to verify the diagnostic applicability of the isolated EVs, and detected mRNA derived from melanoma cells and CD81 in isolated EVs.

## Introduction

The extracellular vesicles (EVs) are nano-sized (50–1000 nm) lipid bilayer sphere that encloses components from their mother cell such as membrane proteins and nucleic acids [1, 2]. Cells secrete EVs continuously, so they exist in most biological fluids [3–5]. These characteristics suggest that EVs may be useful as biomarkers for disease detection, especially tumor detection [6–10]. However, EVs coexist with contaminants such as cellular debris and proteins in the biological fluids; these contaminants interfere with disease detection steps such as sequencing and western blotting. Therefore use of EVs for diagnostic purposes requires a method to effectively eliminate these contaminants.

Extant methods to isolate EVs include ultra-centrifugation, immunoisolation, microfluidics and precipitation in polymeric solution [11]. Ultra-centrifugation is the most conventional method due to its reliability, but it has the demerits of lengthy and laborious centrifugation, need for large starting volume, requirement for expensive equipment, and low yield [12]. Immunoisolation which uses beads conjugated with an antibody to isolate EVs [13]; this method has high specificity, but the EVs are hard to detach from beads, and detachment methods may reduce the functionality of the surface proteins [12]. The process is also expensive to scale up. Microfluidics combined with immunoaffinity requires complex pretreatment and has low throughput [14]. Isolation using the polymeric method based on polymeric precipitation [15] is simple and easy, but requires long incubation time and cannot distinguish EVs from contaminants because it precipitates all of the particles in the sample [16]. To solve these problems, we propose use of an aqueous two-phase system (ATPS) to isolate EVs.

Generally, an ATPS consists of two polymers or a polymer and salt that are immiscible, but in some special cases the process of measurement using X-ray diffraction can cause miscible polymers to become immiscible [17]. Due to the characteristics of ATPSs, such as low interfacial tension, high water content, simple and mild extraction procedure, they have been used as effective tools to extract cells and biomolecules including proteins and antibodies [18]. Moreover it allows concurrent concentration and purification [19]. Isolation of particles by ATPS is based on uneven partitioning of particles between two phases due to the surface properties of particles and the properties of phase system [20]. However, selectivity of partitioning is often inadequate when particles and contaminants have similar surface properties.

Partitioning can be improved by repeating the isolation steps [21–25]. This technique is similar to liquid-liquid chromatography which has a stationary phase and a mobile phase, and which entails repeated re-partitioning between them. For example, a batch procedure that uses polyethylene glycol/dextran (PEG/DEX) ATPS successfully purified plant plasma membrane [26]. In this system, most plasma membranes are partitioned into the PEG-phase, but some of the contaminants are also partitioned into the PEG-phase. To eliminate these contaminants, the PEG-phase is set to be the stationary phase in the first partitioning step, and is re-extracted twice with fresh mobile DEX-phase because the contaminants are partitioned into the DEX-phase while the most of plasma membranes remain in the PEG-phase even during re-extraction.

In this study, the PEG/DEX ATPS was used to isolate EVs from mixture of EVs and serum proteins. To evaluate how polymer concentration affects isolation efficiency, various PEG concentrations were studied. The purity of EVs was increased by applying batch procedure in which DEX-phase is the stationary phase. This method can isolate EVs rapidly from small samples, and does not require any specialized equipment. Therefore it will be helpful in EV-based research and further applications.

## Materials and Methods

### Ethics statement

All animal experiments were approved by the Institutional Animal Care and Use Committee at POSTECH, Pohang, Republic of Korea (approval number: 2013-01-0016). All surgery was performed under avertin (Sigma Aldrich) anesthesia, and all efforts were made to minimize suffering.

### Animal care

All mice used in this study were maintained in the specific pathogen free (SPF) area at the Pohang University of Science and Technology animal facility. Mice were housed in a temperature-controlled container with 12/12 h light/dark cycle with *ad libitum* access to food and water and were monitored daily. When tumors had reached ~2 cm in diameter, they were removed surgically. Before this process, the mice were anesthetized by intraperitoneal injection of avertin. After the tumor tissue was removed, the mice were euthanatized by cervical dislocation.

### Preparation of extracellular vesicles

EVs were isolated from tumor interstitial fluid by ultracentrifugation [27]. C57BL6/j mice were purchased from Jackson laboratory (Bar Harbor, ME, USA). B16BL6 mouse melanoma cells were cultured in minimum essential media alpha (Gibco)- 10% fetal bovine serum (HyClone) containing 1% antibiotics (Gibco). One million such were injected subcutaneously into the basal body of 6-week-old mice to form tumors. The tumor tissues were surgically removed 3 weeks after injection, cut into small pieces and washed with phosphate-buffered saline (PBS) containing 5 mM of ethylenediaminetetraacetic acid (EDTA) on a shaker for 30 min. After washing, the tumor tissues were removed and supernatants which contain interstitial fluid were centrifuged serially at 200×g for 5 min, 500×g for 10 min and 3000×g for 20 min to eliminate cells and cellular debris. Finally cell-free supernatants were ultra-centrifuged at 100,000×g for 2 h. After ultra-centrifugation, the pellet was resuspended in PBS and the quantity of EVs was determined using a Bradford protein assay.

### Preparation of vesicle-free proteins

To represent diverse proteins and other biological factors, bovine calf serum (HyClone) was used as the source of proteins. The serum was heat-inactivated at 56°C for 30 min, then the concentration of native EVs in the serum was depleted by ultra-centrifugation at 150,000×g for 16 h. The amount of protein in the serum was quantified using a Bradford protein assay.

### Preparation of standard samples

To analyze the efficiency of ATPS isolation method, three quantified samples were prepared: 2000 μg/ml standard protein solution; 100 μg/ml standard vesicle solution, and a standard mixture of mixed 2000 μg/ml proteins and 100 μg/ml EVs. Samples were diluted with PBS to desired concentrations.

### Aqueous two-phase system

The phase diagram was determined using turbidometric titration [28]. PEG with molecular weight 25,000~45,000 (Sigma Aldrich) and DEX with molecular weight 450,000~650,000 (Sigma Aldrich) was dissolved in PBS to form two-phase system with a range of compositions, then titrated with PBS until the system just turned clear, which means that a one-phase had formed. The phase transition points were calculated from the weight of added PBS.

Partitioning studies were conducted using polystyrene beads and B16BL6 melanoma cells. The beads and cells were suspended in PEG-phase, then DEX-phase and PEG-phase with particles were introduced into the interface between a slide and a coverslip. Partitioning was observed under a microscope (IX71, Olympus).

An aqueous two-phase system was formed by dissolving PEG and DEX in the standard samples. PEG and DEX were weighed and mixed with 500 μl of standard samples, then stored for 3 h on a shaker (SHO-1D, Wisd Laboratory Instrument) at 200 RPM, 4°C to ensure that the polymers dissolved completely (Table 1). Then the samples were centrifuged at 1000×g for 10 min for phase separation. After phase separation, 310 μl of PEG-phase was collected. To obtain uniform DEX-phase, the interfacial layer (between PEG and DEX-phase) was carefully removed (135 μl, 130 μl and 125 μl was removed in system A, B and C respectively). Then DEX-phase was collected and 50 ul of them was used for further analysis.

**Table 1:** System composition and phase volume of ATPS.

System	System composition (w/w %)		Phase volume (μl)	
	PEG	DEX	PEG	DEX
A	4.5	1.5	445	55
B	4.0	1.5	440	60
C	3.5	1.5	430	70

The batch procedure was performed as an additional step to enhance the purity of extracted EVs. A large phase system (40 g) was prepared by directly dissolving PEG and DEX in PBS in the same composition as in the system used to isolate EVs. After complete dissolving, the solution was centrifuged at 1,000×g for 10 min. Then each phase was collected separately. These fresh PEG and DEX-phases had the same composition as the system which was used to isolate EVs. After the first phase separation, 400 μl of the (top) PEG-phase was carefully removed without touching the interface. Then same volume of fresh PEG-phase was added to the remaining (bottom) DEX-phase and interface, and the sample was mixed vigorously and centrifuged at 1,000×g for 10min. These steps were repeated until the desired number had been completed. In this study, the fresh top phase was transferred twice for Batch number 2, and four times for Batch number 4.

### Isolation of EVs by ultracentrifugation

For comparison, conventional ultra-centrifugation method was performed to isolate EVs from standard mixture: 500 μl of standard mixture was diluted with 65 ml of PBS containing EDTA (final concentration is 5mM) and ultra-centrifuged at 100,000×g for 2 h. After centrifugation, the supernatant was discarded and pellet was dried in the air for 10 min to eliminate liquid. Then the pellet was resuspended in 70 μl of PBS which was the same as the volume of the bottom phase of ATPS used to isolate EVs.

### Quantification of protein

Total protein was quantified using the Bradford method. The bovine serum albumin was used as a standard protein of calibration curve. Absorbance was detected at 595 nm using a microplate reader (DTX 880 Multimode Reader, Beckman Coulter).

### Isolation of RNA

Conventional protein measurement cannot be used to distinguish EVs from serum proteins because EVs membranes include proteins. However, EVs are the only source of RNA in the standard samples, so the quantity of EVs was estimated by measuring the amount of RNA: 40 μl of bottom DEX-phase and ultra-centrifuged sample which were each dissolved in 260 μl of PBS were lysed with 500 μl of Isol-RNA lysis reagent (5 PRIME) for 5 min at room temperature (RT). Also 300 μl of PEG-phase was treated in the same way. After lysis, 100 μl of chloroform (Sigma Aldrich) was added and the mixture was held on ice for 2 min, then centrifuged at 13,500×g for 10 min at 4°C to separate aqueous, interface and organic phase. The aqueous phase that contains the RNA was carefully collected without touching the interface, then an equal volume of isopropanol (IPA) (Sigma Aldrich) was added to precipitate the RNA. After addition of IPA, the samples were held at -20°C for 20 min, then centrifuged at 13,500×g for 10 min at 4°C. The supernatant IPA was discarded and the pellet was washed in 75% of ethanol (Sigma Aldrich) then centrifuged again at 13,500×g for 10 min. The supernatant ethanol was discarded and the pellet was dissolved in 20 μl of nuclease-free water. The amount of RNA was measured using a spectrophotometer (Jenway). Blanks with the same phase composition were measured and their spectra were subtracted from the total spectrum.

### Nanoparticle Tracking Analysis (NTA)

To assess the relationship between the amount of RNA and the number of EVs, NTA was used. RNA quantified samples were placed in the chamber of a Nanosight LM10 (Malvern Instruments Ltd.) and analyzed using Nanoparticle tracking analysis software to count the number of EVs. The number of EVs in ATPS was converted from RNA amount based on this calibration relationship.

### Transmission Electron Microscopy (TEM)

To verify morphology, the isolated EVs were imaged by TEM: 5 μl of each sample was dropped on a formvar carbon film (Electron Microscopy Science, FCF300-cu) for 5 s and removed using filter paper, then 7 μl of 2% uranyl acetate was added and staining was allowed to continue for 10 s and excess was removed using filter paper. The samples were dried in air overnight and imaged at 73 kV acceleration voltage on a transmission electron microscope (JEM-1011, Jeol).

### Western blot

In western blot, the same initial volume of the samples were used for estimating productivity, and the same protein amount of the samples after isolation were used for evaluating purity. Each sample was mixed with 10 μl of 5x SDS loading buffer (250mM Tris-HCl, 10% SDS, 0.5% bromophenol blue, 50% glycerol). The mixed samples were boiled at 100°C for 10 min and run in SDS PAGE (12% resolving gel, 120 V, 90 min). The bands in the gel were transferred to a polyvinylidene difluoride membrane at 390 mA, 2 h, 4°C. The membrane was blocked with 3% non-fat milk (Santa Cruz) in Tri-buffered saline for 1 h at RT and incubated with 0.2 μg/ml of CD81 primary antibody (Santa Cruz, American Hamster Anti-Mouse) in blocking solution at 4°C overnight. Finally, 0.08 μg/ml of horseradish peroxidase-conjugated secondary antibody (Santa Cruz, Anti-Hamster IgG HRP) diluted in blocking solution was attached to the first antibody at RT, and protein bands were detected using a chemiluminescent substrate (West-Zol Plus, iNtRON Biotechnology).

### Reverse transcription-polymerase chain reaction (RT-PCR)

RT-PCR was performed with 4.5 μl of RNA samples. The RNA was reverse transcribed using a reverse transcription kit (GoScript, Promega) and amplified using a polymerase chain reaction kit (GoTaq, Promega) according to the manufacture’s protocol. The primer sequence used in PCR were: Melan A forward 5’-CGCTCCTATGTCACTGCTGA- 3’, reverse 5’-GGTGATCAGGGCTCTCACAT-3’; GAPDH forward 5’-AACACAGTCCATGCCATCAC- 3’ reverse 5’-TCCACCACCCTGTTGCTGTA- 3’. The PCR protocol consisted of denaturation (90°C for 5 min), 40 cycles of amplification (90, 50 and 72°C) for 30 s each and extension (72°C for 10 min). The amplified samples were separated by electrophoresis on 1% agarose gel with SYBR Green DNA staining agent (Invitrogen). The band was imaged using a BioDoc-It imaging system (UVP).

## Results and Discussion

### Surface Effect on Partitioning

**Fig 1:**
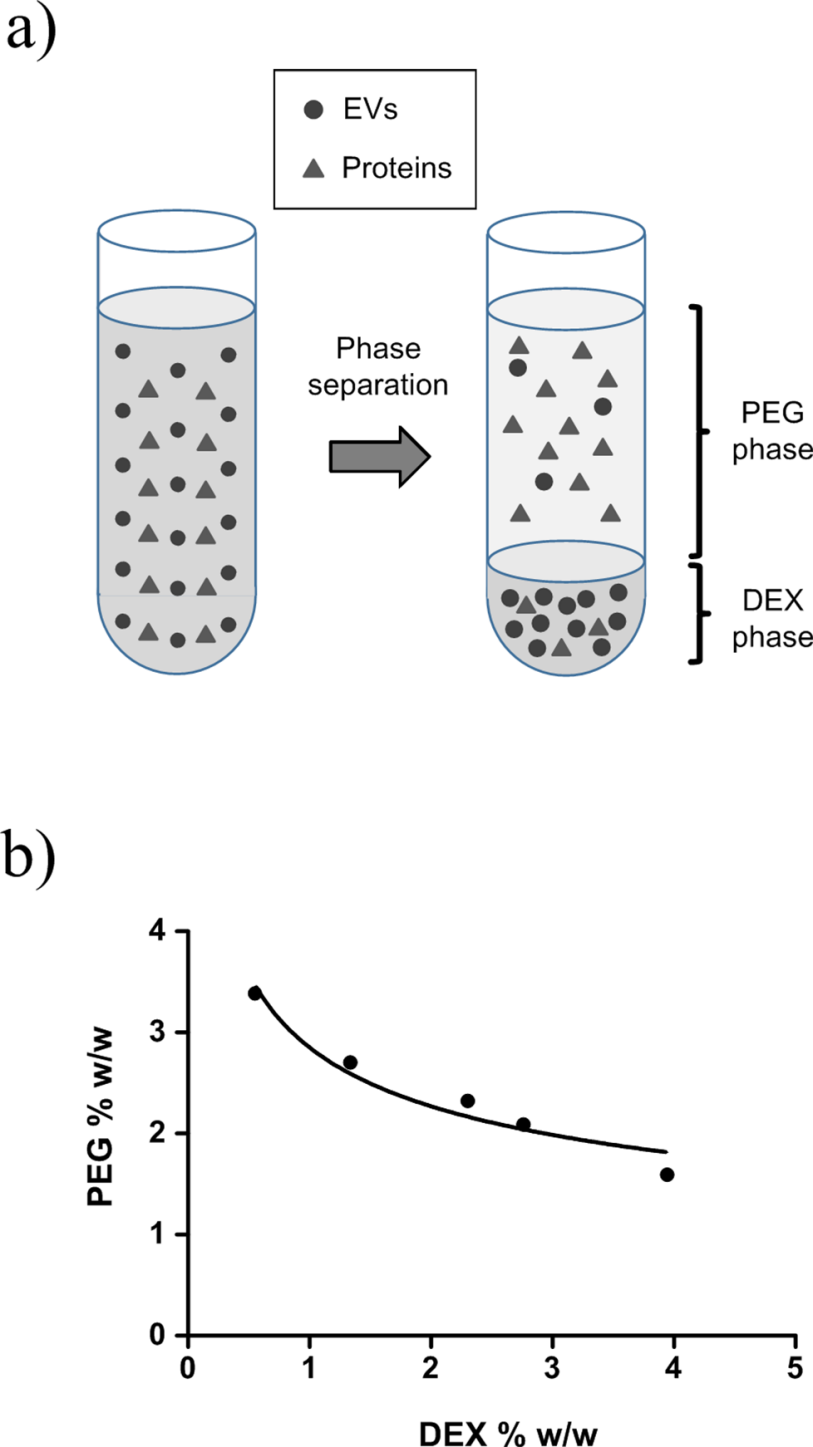
Scheme and phase diagram for PEG/DEX ATPS. (a) Scheme of ATPS separation. EVs prefer DEX-phase to PEG-phase after phase separation using centrifugation (~1000ⅹg). (b) Phase diagram of PEG/DEX ATPS. The two-phase forms when system concentration is above the binodal curve.

In PEG/DEX ATPS, the PEG was enriched in the top phase and DEX was enriched in the bottom phase due to their relative densities (Fig 1A). The PEG concentration at which the transition between two-phase and one-phase system occurred decreased as the proportion of DEX increased (Fig 1B).

**Fig 2:**
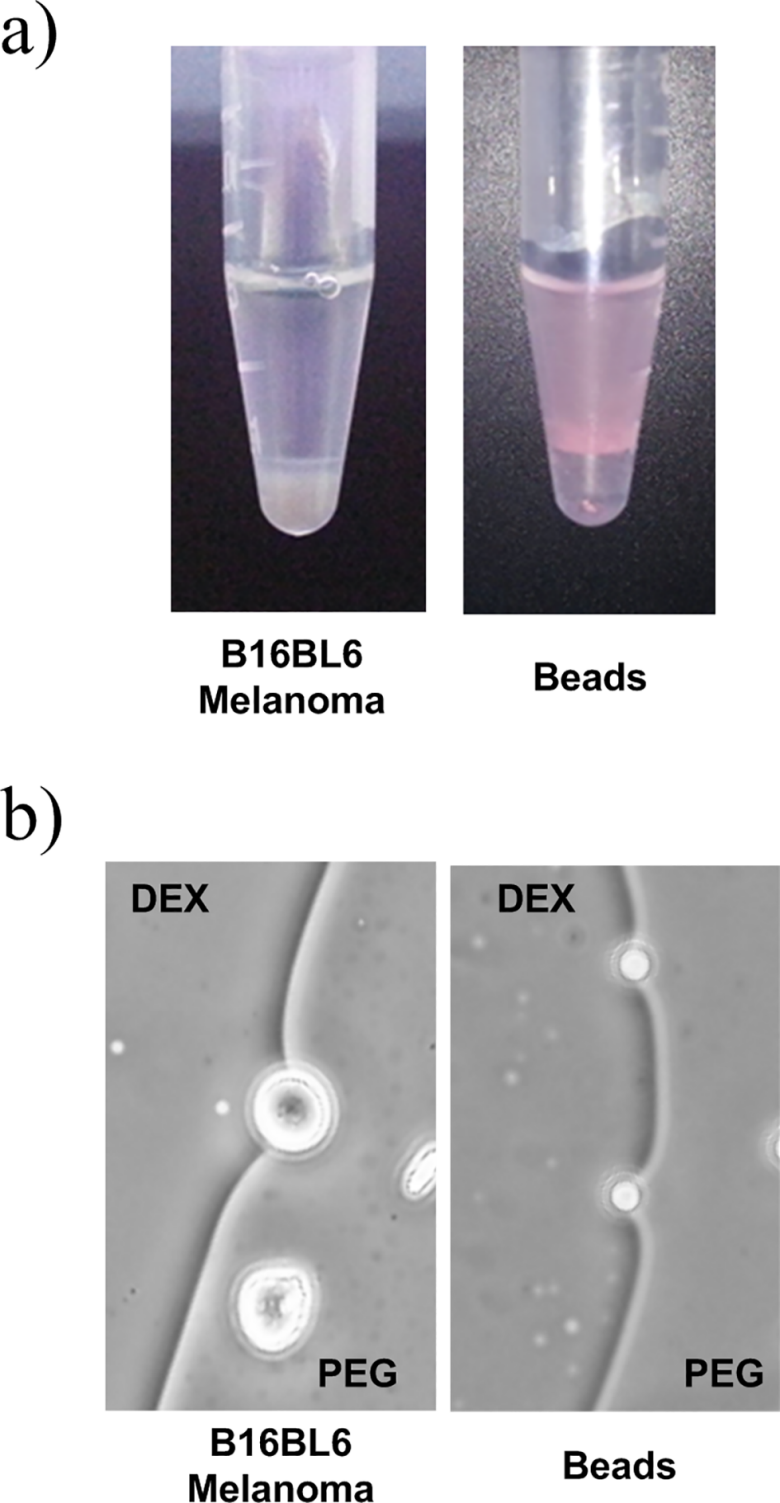
Partitioning studies. (a) Image of partitioned B16BL6 melanoma cells and polystyrene beads (hydrophobic) after phase separation. (b) Contact angle of melanoma and polystyrene beads with DEX-phase. The contact angle between melanoma and DEX-phase was ~40°, indicating that the cell membranes prefer DEX-phase to PEG-phase. Meanwhile, the contact angle between the polystyrene bead and DEX-phase was ~150°, indicating that polystyrene beads prefer PEG-phase.

To investigate the partitioning behavior of a cell membrane that is similar to the EVs membrane, B16BL6 melanoma and polystyrene beads were partitioned using PEG/DEX ATPS (Fig 2A). The beads partitioned preferentially into the top PEG-phase and the B16BL6 partitioned preferentially into the bottom DEX rich phase. The contact angle with DEX-phase were observed when B16BL6 and beads were placed in interface. Due to the difference in their preferences for PEG and DEX, the beads and B16BL6 showed opposite directions of contact angle (Fig 2B). The contact angle between melanoma and DEX-phase was ~40°, which indicates that surface tension between cell membrane and PEG-phase is higher than that between cell membrane and DEX-phase, and that cell membrane partitions more readily into the DEX-phase than into the PEG-phase. In contrast, the contact angle between polystyrene beads and DEX-phase was ~150°, which indicates that polystyrene beads dissolve more readily into the PEG-phase than into the DEX-phase. By the same principle the uneven distribution of particles in ATPS may occur due to the preference of the particle’s surface for one or the other phase. The results imply that EVs would be partitioned into the bottom DEX-phase because the phosphate head in their lipid bilayer membrane has similar surface properties with those of cell membrane of melanoma B16BL6.

### Effect of PEG concentration on partitioning of EVs and proteins

To quantify the degree of isolation, the partition coefficient *K*_*Prot*_ of protein was calculated as

$$K_{\text{Prot}}=\frac{C_{\text{top}}}{C_{\text{bottom}}}\;,$$ (1)

where *C*_*top*_ and *C*_*bottom*_ represent the concentration of proteins in the top and bottom phase, respectively.

**Fig 3:**
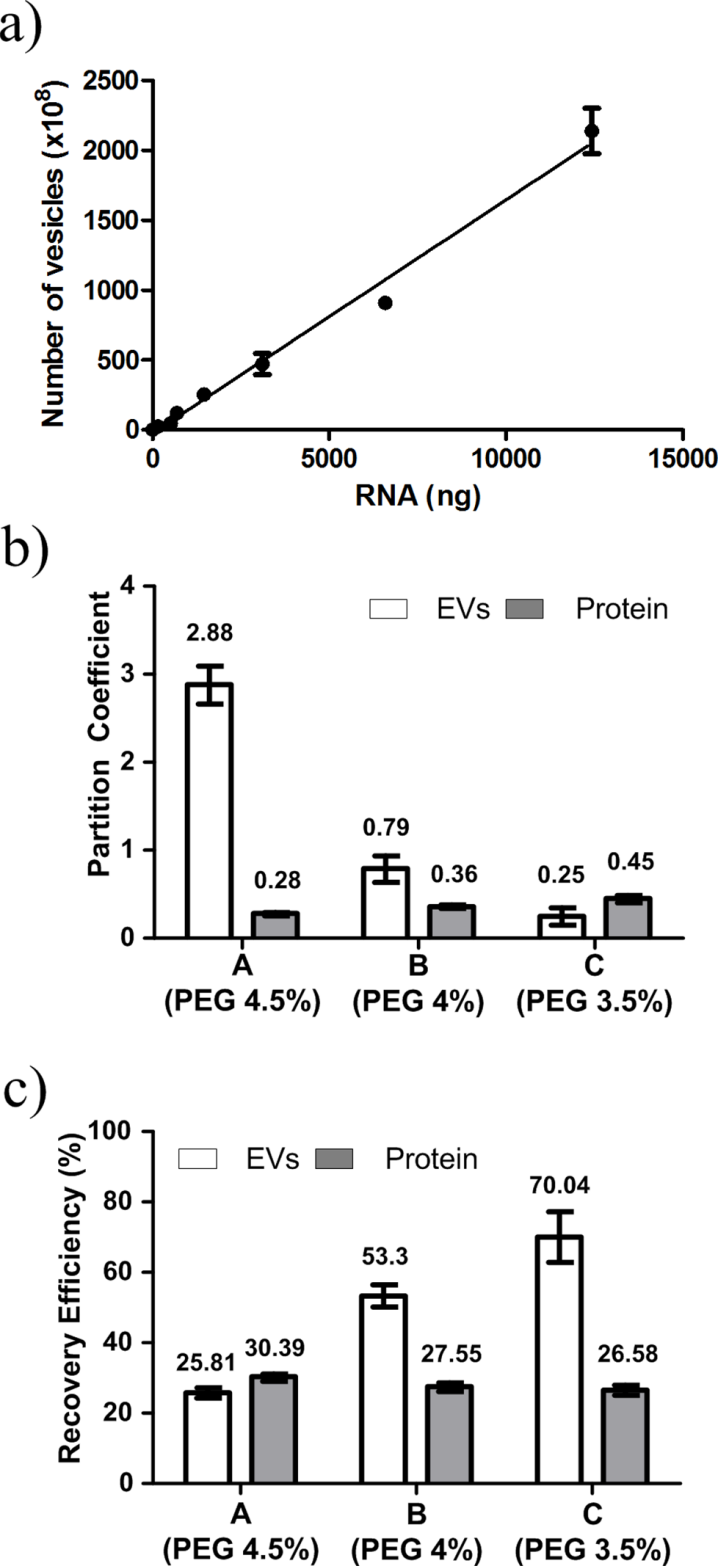
Effect of PEG concentration in partitioning of EVs and proteins. (a) Relationship between RNA amount and number of EVs. (b) Partition coefficient *K*. (c) Recovery efficiency: amount in DEX-phase relative to total amount.

In contrast to the proteins, which are soluble, particles such as cells or EVs are partitioned in one bulk phase and at the interface between the two phases [18, 20, 29]. In PEG/DEX ATPS, the most of EVs were partitioned in the bottom DEX-phase (S1 Fig). Therefore, the partition coefficient *K*_*EVs*_ of EVs was calculated as

$$K_{EVs}=\frac{N_{\text{int}}}{N_{\text{bottom}}}\;,$$ (2)

where *N*_*int*_ and *N*_*bottom*_ represent the number of EVs in the interface and the bottom phase, respectively. The number of EVs was converted from quantified RNA amount using the calibration curve (Fig 3A). The recovery efficiency that describes yield in the bottom phase was calculated as

$$\text{Recovery efficiency}=\frac{Amount\;of\;particles\;in\;the\;bottom\;phase}{Total\;particles\;amount\;in\;system}\;.$$ (3)

The level of enrichment of protein and EVs were described as

$$\text{Enrichment ratio}=\frac{C_{bottom}}{C_{Total}}=\frac{N_{bottom}}{N_{\text{total}}}\;,$$ (4)

where *C*_*Total*_ and *N*_*Total*_ represent the concentration of proteins and number of EVs in the total system respectively.

To assess the partitioning behavior of EVs and proteins without interactions between them, standard samples of proteins (2000 μg/ml) and EVs (100 μg/ml) were partitioned individually. Then the mixture of serum protein and EVs was used to simulate biological fluid. To optimize the polymer concentration for isolation of EVs in ATPS, three PEG concentrations were used (Table 1). As PEG concentration decreased, *K*_*prot*_ increased from 0.28 to 0.45, and *K*_*EVs*_ decreased from 2.88 to 0.25 (Fig 3B). The *K*_*prot*_ < 1 means that the proteins were more concentrated in the DEX-phase than in the PEG-phase and *K*_*EVs*_ < 1 means that the EVs were preferentially partitioned in the DEX-phase rather than in the interface. The partitioning results indicated that the EVs were increasingly partitioned into the DEX-phase while partitioning of protein in DEX-phase was decreased as PEG concentration decreased. This opposite trend can be explained by the effect of the interfacial tension between top PEG-phase and bottom DEX-phase. High interfacial tension can trap particles at the interface regardless of their preference for phase. This tension is proportional to the polymer concentration of the system; as PEG concentration decreased, the interfacial tension decreased, so the number of EVs trapped in the interface decreased [18, 20, 30]. As PEG concentration decreased, the recovery efficiency of proteins was not affected but the recovery efficiency of EVs increased from ~30% to ~70% (Fig 3C).

**Fig 4:**
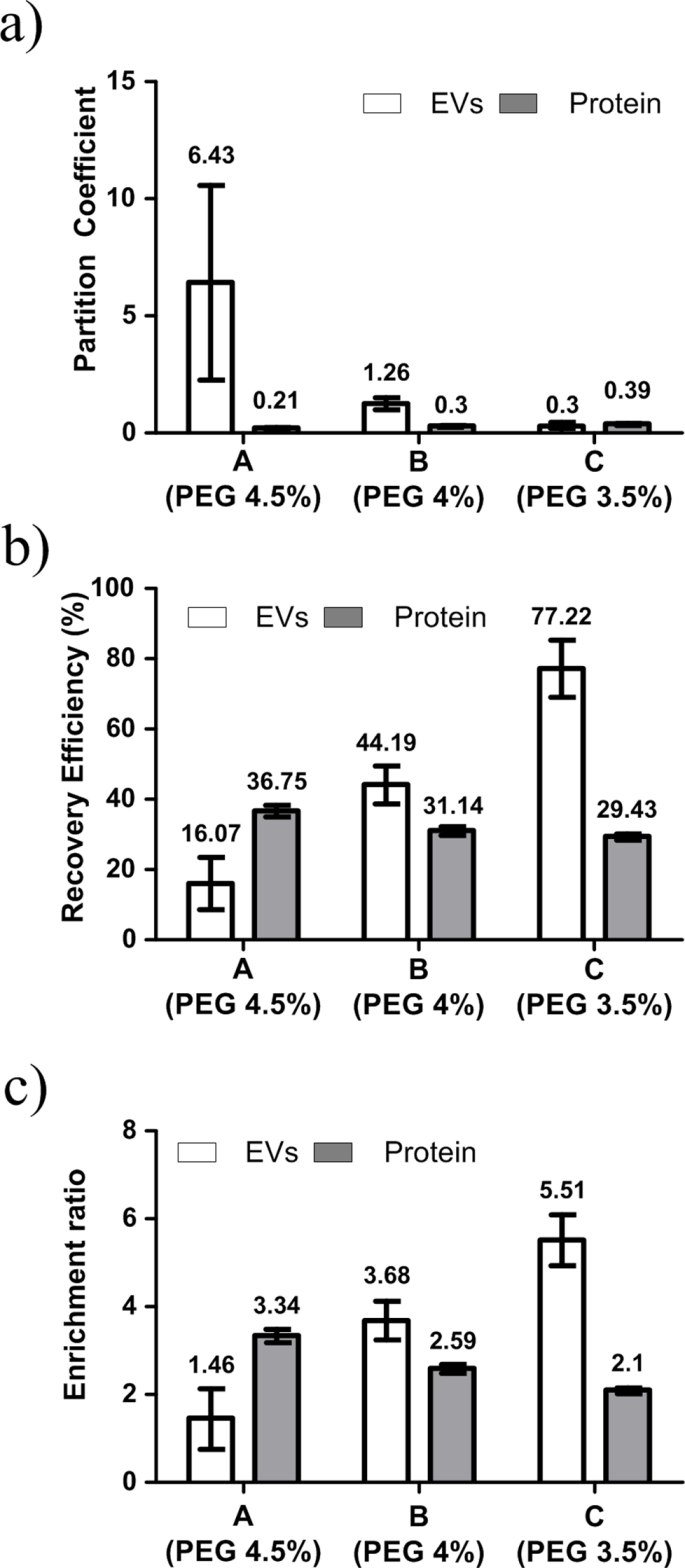
Effect of PEG concentration in partitioning of mixture of EVs and proteins. (a) Partition coefficient *K*. (b) Recovery efficiency: amount in DEX-phase relative to total amount. (c) Enrichment ratio: concentration in DEX-phase relative to initial concentration.

Partitioning of a mixture of EVs and serum proteins was investigated using a standard mixture (EVs: 100 μg/ml, proteins: 2000 μg/ml). As in the case of pure EVs and proteins, *K*_*Prot*_ increased and *K*_*EVs*_ decreased as PEG concentration decreased (Fig 4A). These results indicate that the low PEG concentration is suitable for eliminating proteins to increase the purity of EVs. The recovery efficiency showed a similar trend. The highest recovery efficiency of EVs and lowest recovery efficiency of proteins were obtained at the lowest PEG concentration (Fig 4B). Additionally, EVs were concentrated at the same time. Due to the small volume of the DEX-phase and the high recovery efficiency, EVs were concentrated in the bottom DEX-phase. The enrichment ratio indicated that system C had 5 times higher EVs concentration than the standard mixture (Fig 4C).

Considering these results using both pure and mixed proteins and EVs, we selected system C for use in isolation of EVs because it gave the highest recovery efficiency and the highest enrichment ratio.

### Purification of EVs by batch procedure

**Fig 5:**
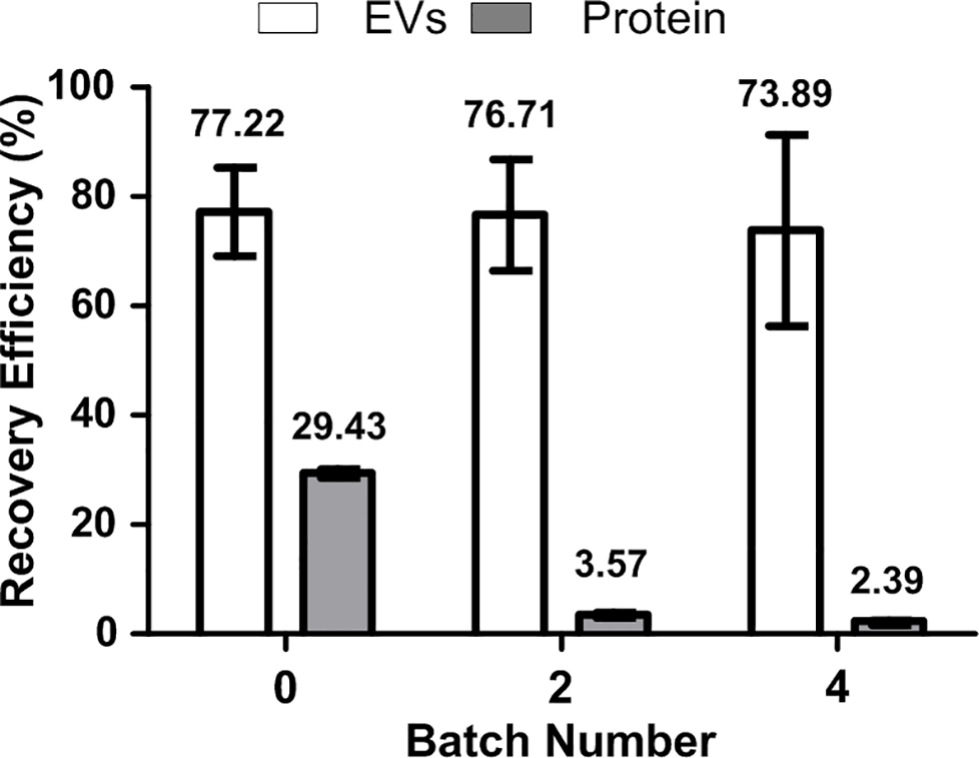
Recovery efficiency of EVs and proteins in batch procedure. After batch procedure, the recovery efficiency of EVs was almost unchanged while the recovery efficiency of proteins decreased.

Although the lower PEG concentration of system was suitable for EVs isolation, ATPS concentrates not only EVs but also proteins. To increase the purity of EVs, the batch procedure was performed to eliminate remaining serum proteins from the bottom DEX-phase. Repeated replacement of the PEG-phase by fresh PEG-phase extracted more proteins than EVs from the DEX-phase because most of the EVs were partitioned into the interface and bottom DEX-phase whereas only ~30% of proteins remained in the bottom DEX-phase. After replacing the PEG-phase four times, the amount of proteins in the bottom DEX-phase decreased to one tenth of that in the single ATPS, but the quantity of EVs was nearly unchanged (Fig 5).

### Comparison with ultra-centrifugation method for analysis

**Fig 6:**
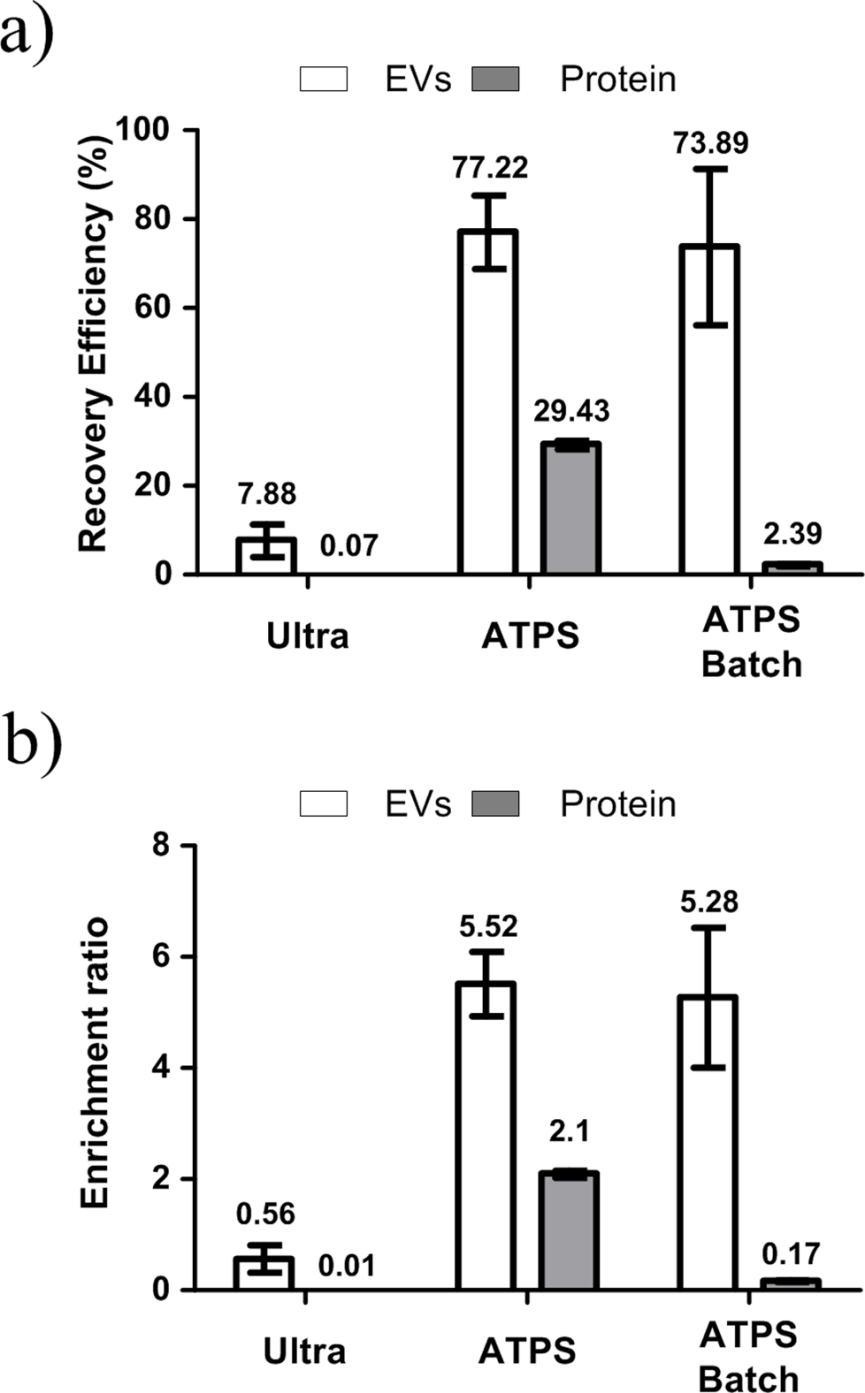
Comparison of ATPS, ATPS-Batch #4 and ultra-centrifugation method. (a) Recovery efficiency. (b) Enrichment ratio.

To evaluate the ATPS isolation method, its recovery efficiency and purity of EVs were compared with those obtained using conventional ultra-centrifugation. The recovery efficiencies of ATPS and ATPS combined with a batch procedure (ATPS-batch) were seven times higher than those of ultra-centrifugation (Fig 6A). Moreover, due to the small volume of the bottom DEX-phase, ATPS and ATPS-batch increased the concentration of EVs to five times than the initial concentration, whereas ultra-centrifugation method reduced it (Fig 6B).

**Fig 7:**
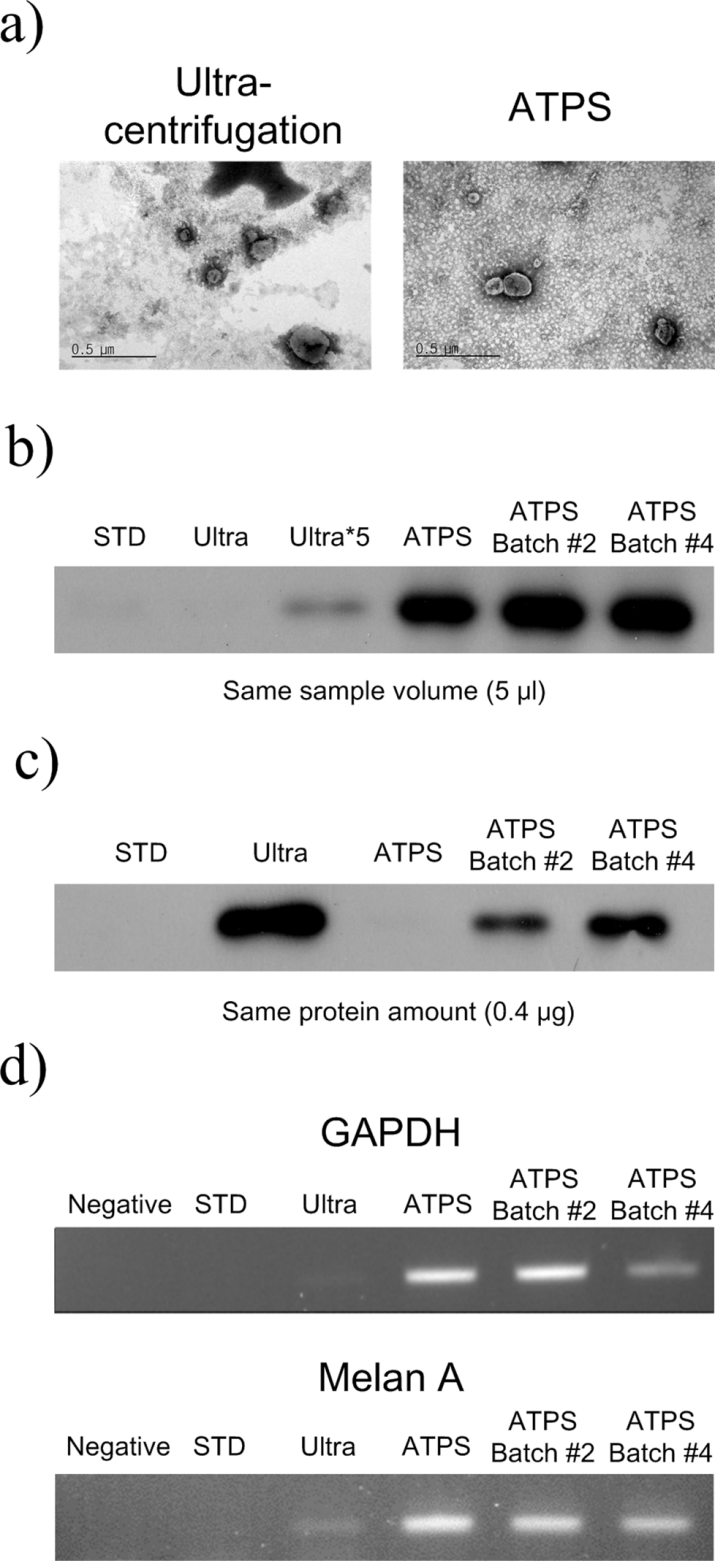
TEM, western blot and RT-PCR for comparison of ATPS, ATPS-Batch and ultra-centrifugation. (a) TEM image of EVs from ATPS and ultra-centrifugation method. The image did not show morphological difference between both methods. (b) The pellet after ultra-centrifugation was resuspended in 70 μl of PBS which was the same as the volume of the bottom phase of ATPS used to isolate EVs. Using the prepared samples, CD81 western blot was performed for the same sample volume (5 μl). Protein samples (5 μl) from standard mixture, ultra-centrifugation (25 μl for Ultra*5), ATPS method, and ATPS combined with Batch number 2 and 4 (ATPS-Batch #2 and #4) were used to confirm recovery efficiency. The band was brighter than that of the ultra-centrifugation method. (c) Purity of EVs was analyzed by western blot using CD81 antibody with the same protein amount (0.4 μg) from standard mixture, ultra-centrifugation, ATPS method, and ATPS-Batch #2 and #4. (d) RT-PCR was performed with 4.5 μl of isolated RNA from ultra-centrifugation, ATPS and ATPS combined with batch procedure. Bands of the house-keeping gene GAPDH and melanoma tumor marker Melan A were stronger after ATPS and ATPS-Batch than after ultra-centrifugation.

The isolated EVs were morphologically verified, and the EVs from the ATPS and ultra-centrifugation method were imaged using TEM (Fig 7A); EVs obtained using ATPS had similar morphology and size to those obtained by ultra-centrifugation, and all had intact lipid membranes.

To assess the applicability of the isolated EVs and the recovery efficiency of each method, the isolated EVs from the different isolation methods were compared by western blot analysis using specific CD81 marker with the same volume of each sample (Fig 7B). The bands of ATPS and ATPS-Batch were significantly brighter than that of ultra-centrifugation method, even when the volume of sample from ultracentrifugation was increased by a factor of five. To analyze the purity of EVs, the same amount of protein was used (Fig 7C). The brightness of the CD81 bands increased as the number of PEG replacements increased; this trend indicates that the relative amount of EVs-specific protein among total proteins increased as the purity of EVs improved.

Due to the short life of free RNA, the most of the isolated RNA must come from the EVs because their lipid bilayers protect RNA from RNase [31]. To confirm that ATPS is suitable for RNA analysis, RT-PCR was performed to detect Melan A from the isolated EVs, which originated from melanoma cells (Fig 7D). The EVs isolated by ATPS and ATPS-Batch had stronger intensity of bands than did ultra-centrifuged EVs. This result indicates that the isolation methods using ATPS and ATPS-Batch can also be used for RNA analysis.

## Conclusion

This research demonstrated a simple and fast isolation method using PEG/DEX aqueous two-phase system from small volume of sample without any specialized equipment. Compared to conventional ultra-centrifugation method, ATPS isolation method had seven times higher recovery efficiency, and ATPS combined with a batch procedure could increase the purity of the isolated EVs. The diagnostic applicability of ATPS method was confirmed by performing western blot and RT-PCR. This easy and rapid isolation method may help researchers to isolate EVs and to analyze them for diagnostic and prognostic purposes.

## Supporting Information

S1 Fig Distribution of extracellular vesicles in PEG/DEX ATPS. The most of EVs was distributed in DEX and interface.
(TIF)

S2 Fig Original Uncropped image of Fig 7B. Lane 1 is negative control, 2 is Standard mixture, 3 is ultra-centrifugation method, 4 is ultracentrifugation method with five of factor, 5 is ATPS method, 6 is ATPS-Batch #2 and 7 is ATPS-Batch #4.
(TIF)

S3 Fig Original Uncropped image of Fig 7C. Lane 1 is negative control, 2 is Standard mixture, 3 is ultra-centrifugation method, 4 is ATPS method, 5 is ATPS-Batch #2 and 6 is ATPS-Batch #4.
(TIF)

S4 Fig Original Uncropped image of Fig 7D. a) GAPDH, b) Melan A, Lane 1 is negative control, 2 is Standard mixture, 3 is ultra-centrifugation method, 4 is ATPS method, 5 is ATPS-Batch #2 and 6 is ATPS-Batch #4.
(TIF)
