# Supplementary figures and images for: Isolation of High-Purity Extracellular Vesicles by Extracting Proteins Using Aqueous Two-Phase System
Source: PLoS One. 2015 Jun 19;10(6):e0129760. doi: 10.1371/journal.pone.0129760 (PMC4475045; doi:10.1371/journal.pone.0129760)

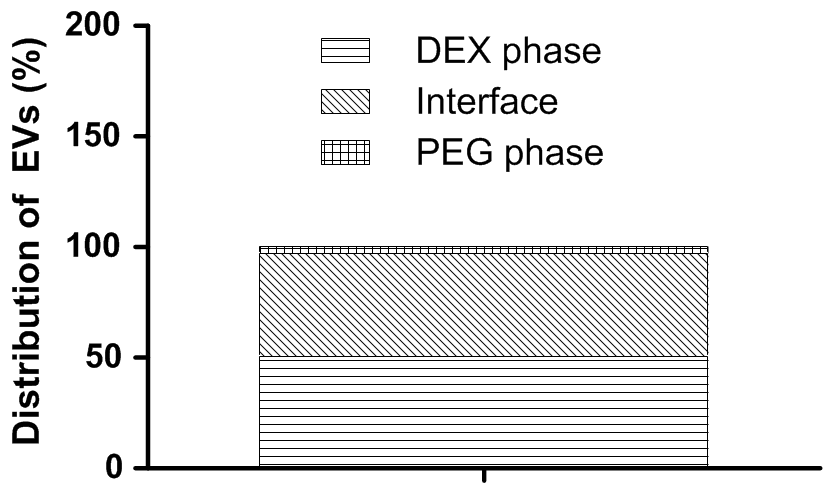

Supplement: S1 Fig — The most of EVs was distributed in DEX and interface. (TIF) [file pone.0129760.s001.tif]

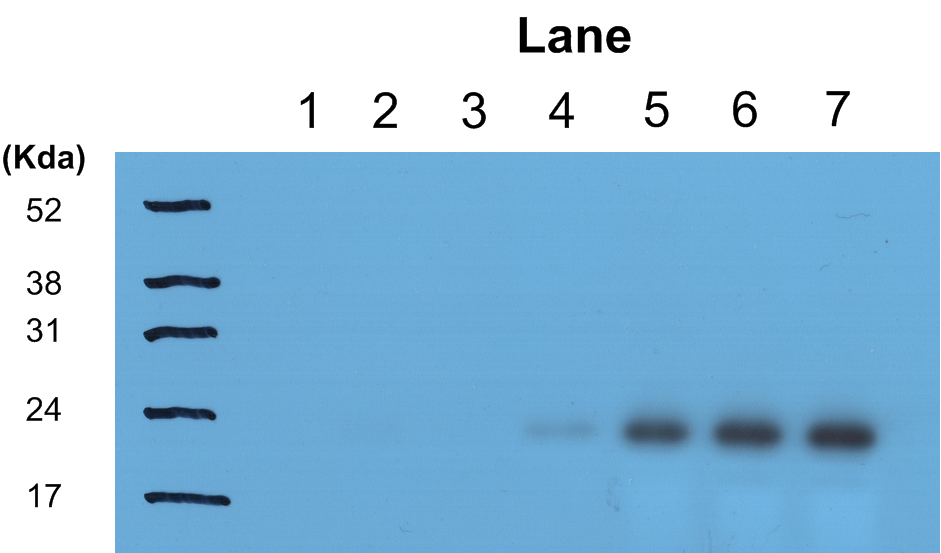

Supplement: S2 Fig — Lane 1 is negative control, 2 is Standard mixture, 3 is ultra-centrifugation method, 4 is ultracentrifugation method with five of factor, 5 is ATPS method, 6 is ATPS-Batch #2 and 7 is ATPS-Batch #4. (TIF) [file pone.0129760.s002.tif]

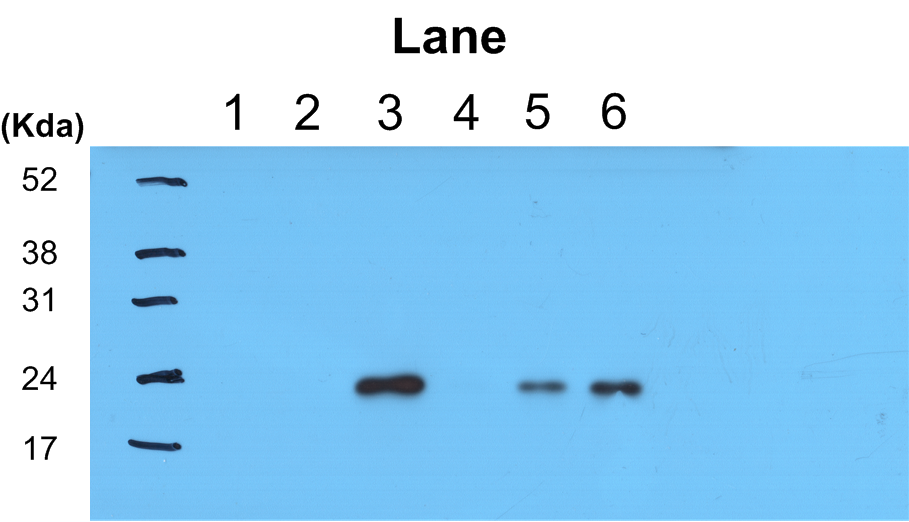

Supplement: S3 Fig — Lane 1 is negative control, 2 is Standard mixture, 3 is ultra-centrifugation method, 4 is ATPS method, 5 is ATPS-Batch #2 and 6 is ATPS-Batch #4. (TIF) [file pone.0129760.s003.tif]

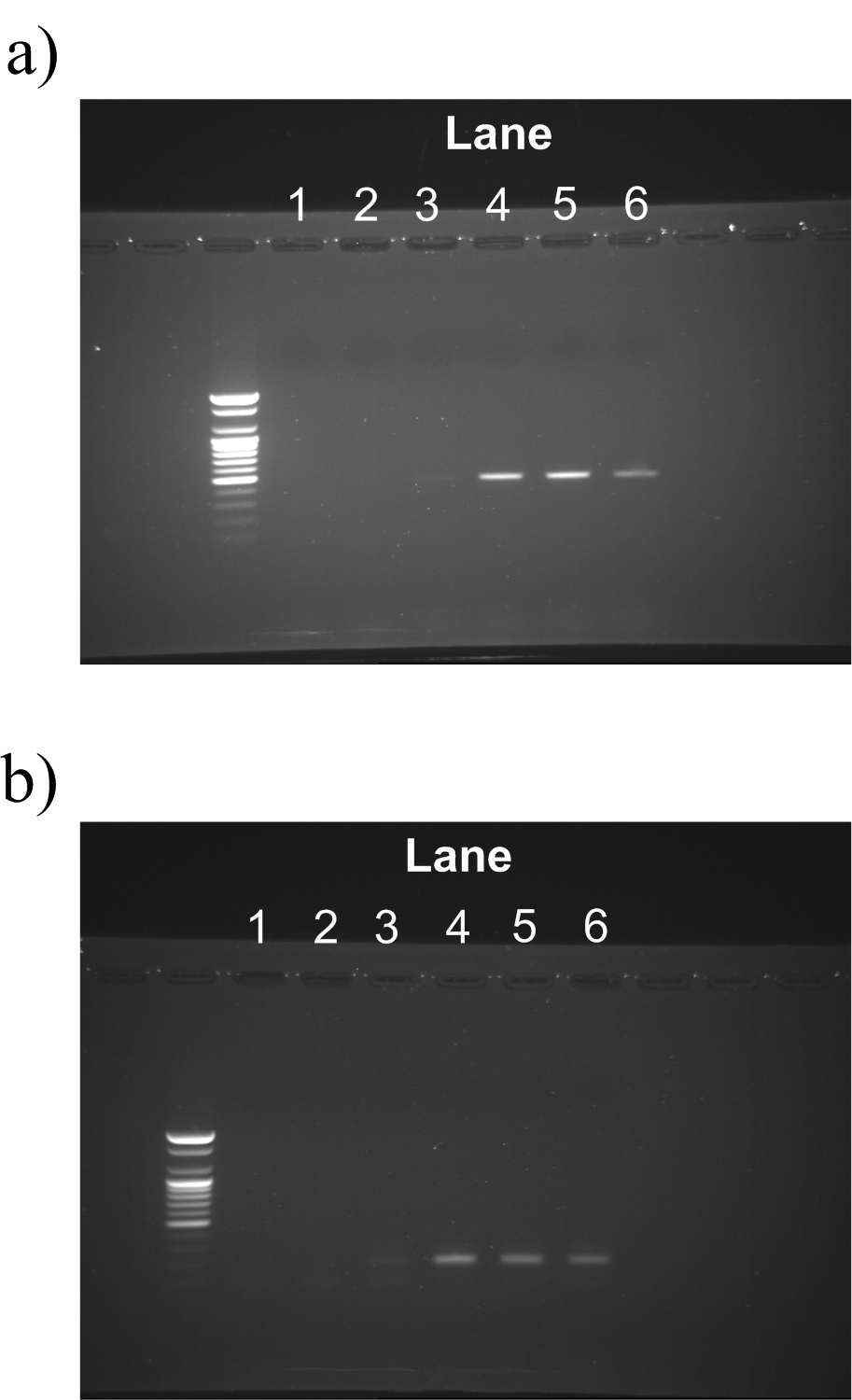

Supplement: S4 Fig — a) GAPDH, b) Melan A, Lane 1 is negative control, 2 is Standard mixture, 3 is ultra-centrifugation method, 4 is ATPS method, 5 is ATPS-Batch #2 and 6 is ATPS-Batch #4. (TIF) [file pone.0129760.s004.tif]
